# Facts and Challenges about Asthma and COVID-19 among the Paediatric Population: A Systematic Literature Review

**DOI:** 10.3390/medicina57121306

**Published:** 2021-11-29

**Authors:** Emilia Moreno-Sánchez, Estefanía Castillo-Viera, Emilia Vélez-Moreno, Francisco-Javier Gago-Valiente

**Affiliations:** 1 Department of Pedagogy, Faculty of Education, Psychology and Sports Sciences, University of Huelva, 21071 Huelva, Spain; emilia@uhu.es; 2 Faculty of Education, Psychology and Sports Sciences, University of Huelva, 21071 Huelva, Spain; 3 Medicine Department, Faculty of Medicine, University of Malaga, 29071 Malaga, Spain; eliavm3@gmail.com; 4 Preventive Medicine and Public Health Area, Faculty of Labour Sciences, University of Huelva, 21071 Huelva, Spain; francisco.gago@dstso.uhu.es

**Keywords:** asthma, coronavirus, COVID-19, SARS-Cov-2, paediatric population, paediatrics

## Abstract

A systematic review of the literature was conducted to analyse the factors that affect the probability of the paediatric asthma population suffering from COVID-19 or SARS-CoV-2, such as asthma phenotypes, inhaled corticosteroids, and the effects of lockdown. This systematic review was based on PRISMA guidelines. A bibliographic search was conducted using BNE, BVS (LILAC), CSIC (IME, ISOC), IBECS, Scielo, Scopus, Medline, and PubMed, using the following search profile: (COVID-19 or 2019-NCOV or SARS-CoV-2 or COV-19) AND asthma AND (children or adolescents or youths or children or teenagers). The results were limited to those articles published between December 2019 and December 2020, selecting only articles published in Spanish, English and French that included the study population (children aged 0–18 years). Among the 1066 results of the bibliographic search and seven articles selected from a manual search, only 19 articles were found to fit our eligibility criteria. Most of the articles highlight the effects of lockdown on the paediatric asthma population, increased therapeutic compliance, and the role of inhaled corticosteroids and intrinsic factors such as ACE2 receptors as causes of the decreased prevalence of COVID-19 among the paediatric asthma population. This population has unique characteristics that serve as protective factors against COVID-19. The safety measures implemented during the lockdown period along with inhaled corticosteroid treatment also contributed to this protection.

## 1. Introduction

Coronaviruses are viruses that mainly cause respiratory and gastrointestinal infections and are also classified into four groups: alphacoronavirus, betacoronavirus, gammacoronavirus, and deltacoronavirus. Previously, only two coronaviruses were known: SARS-CoV (discovered in 2002 in Guangdong, China) and MERS (discovered in 2012 in Saudi Arabia). The SARS-CoV-2 virus has been genetically determined and sequenced [1]. The intermediary host, possibly another mammal, has not yet been determined. The site of contact with people might have been a Wuhan live animal market [1,2]. It is proven that this virus is transmitted effectively from person to person, with groups of cases within the family and friends having been found. The most common form of transmission of the virus is by respiratory droplets (aerosols), in enclosed spaces (1.5 m) and by materials contaminated by these aerosols. However, the likelihood of transmission by air cannot be ruled out. Many of the contagions are caused by patients with symptoms, but people who are asymptomatic or in the incubation period can also transmit the disease [3,4]. In December 2019, SARS-CoV-2 was discovered in Wuhan, China. Although it began as a mild disease, it has already caused 160,813,869 cases of COVID-19, including 3,339,002 deaths, reported to the WHO [5]. The most common symptoms of the infection caused by this virus transmitted through droplets are fever, dry cough, and tiredness [6]. However, children appear to present a milder course and may even have no symptoms. This virus uses ACE2 receptors, present in pneumocytes and lung macrophages in lower respiratory tract tissues, to enter the body and cause an infection [7]. Asthma is the most prevalent chronic respiratory disease worldwide [8]. It is suffered by 10% of the Spanish paediatric population and can be defined as a syndrome manifested in patients presenting recurrent symptoms (such as noisy and difficult breathing, chest tightness and cough) caused by lower airway obstruction [9]. These symptoms vary in time and intensity and result from various underlying aetiopathogenic mechanisms [9]. Although it is difficult to establish many of the aetiopathological mechanisms that originate asthma and the moment in which it appears, there are epidemiological studies that mention that the fact that the father or mother suffers from asthma and allergen sensitization are considered as risk factors for suffering from asthma [10]. Nevertheless, we cannot deny the role of the environmental factor in addition to those already cited. Therefore, exposure to tobacco and environmental pollution, especially during intrauterine development, are deemed risk factors [11]. Other factors are related to lifestyle, diet (the type of diet and obesity also increase the frequency and severity of asthma), hygiene (contact with particles and substances of animal and microbial origin) [12]. Asthma is a disease in which viral infections, especially respiratory syncytial virus, and rhinovirus, have a special incidence [13]. This predisposition makes it a priority health challenge to consider the incidence of the SARS-CoV-2 virus (severe acute respiratory syndrome coronavirus 2), which can cause complicated conditions in the asthmatic population. Since the SARS-CoV-2 pandemic began, a milder course of SARS-CoV-2 disease has been reported in the paediatric and juvenile population [14]. In contrast, those with chronic obstructive pulmonary disease or asthma have been considered to have risk factors that favour a more severe and even lethal form of COVID-19 disease [15]. These considerations have also created limitations concerning these people’s lifestyle, leading to an even tougher time for them during lockdown. Meanwhile, statistics on the prevalence of SARS-CoV-2 have appeared worldwide, in which the paediatric asthmatic population is underrepresented [16]. These statistics raise the possibility that this population is considered protected and, therefore, this work aimed to analyse the current scientific evidence available to shed light on this issue. To this end, a systematic review was conducted to analyse the intrinsic biological and social factors of children with this type of pathology, all of which are of considerable importance in this issue.

## 2. Materials and Methods

We conducted a systematic review of the scientific literature on the characteristics that protect asthmatic children and adolescents against SARS-CoV-2 infection. The PRISMA statement criteria for systematic reviews were applied [17]. We applied these criteria to carry out our research in order to analyse the selected articles exhaustively. This study is registered in PROSPERO (International prospective register of systematic reviews) with registration number 245591.

### 2.1. Eligibility Criteria 

Two literature search processes were applied: one conducted in October 2020 and another in November 2020 (with the latter being the focus of the review, as it yielded more results than the former). The search was limited to papers published from December 2019 to December 2020 to avoid results from earlier coronaviruses. Moreover, the search was limited to English, Spanish, or French articles due to the authors’ command of these languages. Finally, it should be noted that we selected only those articles that met our eligibility criteria: they included the study population (age 0–18 years), they were conducted in highincome and uppermiddleincome countries, in addition to the words asthma and COVID or coronavirus in the descriptors.

### 2.2. Information Sources

The databases used for this search were: BNE, BVS (LILAC), CSIC (IME, ISOC), IBECS, Scielo, Scopus, Medline, and PubMed. The search profile used was: (COVID-19 or 2019-NCOV or SARS-CoV-2 or COV-19) AND asthma AND (children or adolescents or youth or child or teenager), to which the above temporal, language and age filters were added. All articles meeting these criteria were selected regardless of the type of study conducted. An additional manual search was conducted to supplement the data obtained.

### 2.3. Study Selection and Data Extraction Process

As stated in the PRISMA statement for improving systematic reviews and meta-analyses, this complex process involved numerous judgements and actions following the search [17]. First, all relevant results were carefully reviewed by analysing the title, abstract, method and conclusions to select the included articles with more certainty. For data extraction, a table was designed with the following items: date of publication, country, type of study, and variables analysed/objectives and results to minimise bias in the review process. To avoid biased opinions, two researchers from the research team with vast experience on the subject and in research methodology intervened [18].

In order to reduce selection bias, each manuscript was independently reviewed by three authors of this work, who decided whether or not a paper met the inclusion criteria. In case there was no consensus on the inclusion or not of any study, the dilemma was solved by consulting the fourth author [19].

## 3. Results

### 3.1. Study Selection

**Figure 1:**
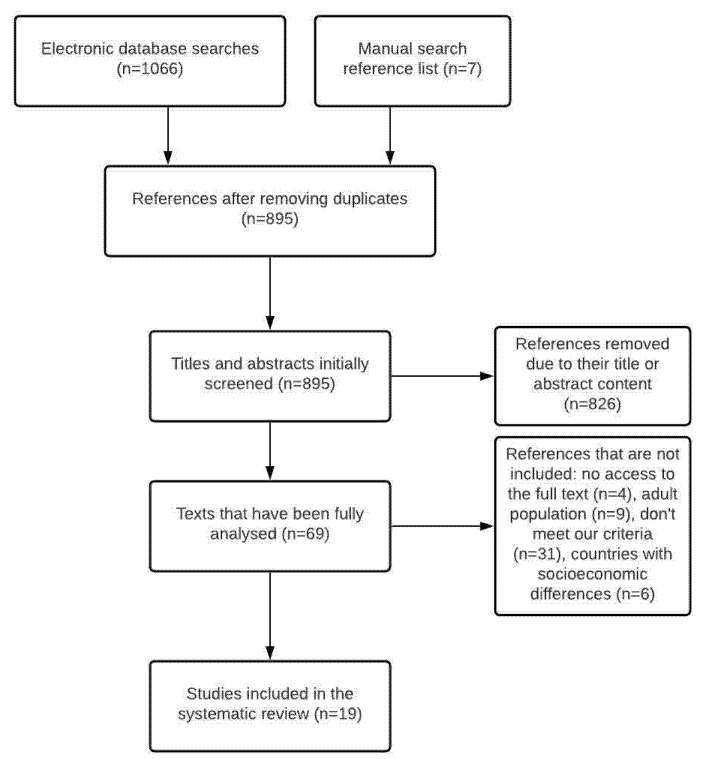
PRISMA flow diagram.

Figure 1 shows the search process followed from the outset, showing the number of studies found and ending with those included in the review.

Following the criteria established in the previous section, 1066 results were obtained from the database search and seven from the manual search. After eliminating duplicates, a total of 895 articles remained. These results were filtered, after which 826 were eliminated because their title or abstract did not meet the review’s objectives. The remaining 69 articles were analysed in their entirety to assess whether or not they met the established eligibility criteria. Consequently, four were rejected because we did not have access to the full texts, nine were excluded because the study population only included adults, 31 did not fit with our study objective, and a further six were conducted in specific socio-economic contexts, which is to say that they were conducted in low- and middle-income countries. A total of 19 articles were selected for review. 

### 3.2. Characteristics of the Studies 

Table 1 shows the characteristics of the 19 articles chosen for the systematic review.

### 3.3. Decrease in Prevalence of COVID-19 among Paediatric Asthmatic Patients

Since the COVID-19 pandemic began, many studies have highlighted the fact that paediatric asthmatic patients have a reduced prevalence of COVID-19 among them. Krivec et al. show that during the 2020 confinement period, there was a 71% to 78% decrease in the number of admissions of paediatric asthmatic patients to at the Ljubljana Children’s Hospital, compared with the same period three years previously [20]. A study carried out in Spain reveals that the percentage of asthmatics admitted up to 30 June 2020 (3.2%) was lower than the estimated prevalence for that territory (6%) [21]. In Italy, at south Lombardy and Liguria Hospitals, only one out of 52 paediatrics patients only one had asthma [11]. Also, an Italian study, the Confidence Study, shows that none of the 100 patients with COVID were asthmatic [38]. Studies from China also show that among all the paediatric patients with COVID infection, none of them were asthmatic [39,40]. Furthermore, a 90% decrease in asthma admissions has been seen in this group at the St George’s University Hospitals NHS Foundation Trust in the United Kingdom [26].At Massachusetts General Hospital a decrease in the number of emergency department visits by asthmatic patients of 39.8% and 84.4% during the months of March and April 2020 has been noticed [25], even though some papers from the US show that asthma is the most frequent comorbidity among children [41]. However, some authors acknowledge that this decrease in prevalence is due to low recognition of chronic lung diseases in patients with COVID-19, produced by these patients’ lack of symptom reporting and the lack of recognition by professionals [22,23]. This hypothesis is rejected in one of the studies analysed, showing that the prevalence of these diseases is zero in many studies, unlike other chronic disorders such as diabetes [22,23]. Another article also rejects this hypothesis, arguing that this low prevalence of asthmatics has been present since the beginning of the pandemic, so this could not be the only cause [27]. To conclude this section, as we can see, many studies show us real data about this decrease in prevalence of COVID-19 among this group of patients. Table 2 shows the data mentioned in this section.

### 3.4. Effects of Confinement

Among school children, most asthma exacerbations are triggered by viruses (85%) [20,22,30]. However, the most frequent cause of exacerbations across all continents is rhinovirus and not coronavirus (responsible for 8.4% only) [21,22,30]. Another possible cause of asthma exacerbation is air pollution, and in this case, it has been shown that improved air quality could lead to a decrease in hospital admissions [20,25,26]. In short, these data show that compliance with safety measures and not being able to attend school has had a positive effect on the health of these children [20,25,27,29,31]. However, one of the studies suggests that the decrease in air pollution could increase the severity of viral exacerbations and, in two studies, it is shown that staying indoors for a long time is also a significant cause of asthmatic exacerbations due to tobacco exposure [37], which can worsen the prognosis of asthma patients with COVID-19, although this association remains clear [25,28,32].

Moreover, it is shown that, following adequate treatment measures, infants do not need to follow other extreme safety measures due to their pathology, as in many cases, this could cause stress and psychological damage [29,33,34]. Several studies have highlighted the role of socio-demographic factors, with children of low socio-demographic status being at most risk for SARS-CoV-2 [25,27]. Specifically, malnutrition is a cause of increased viral transmission in infants because it compromises the immune response [34].

### 3.5. Characteristics of the Paediatric Asthmatic Population 

Asthmatic children have less aggressive forms of SARS-CoV-2 disease, with fever being one of the most frequent symptoms [36]. Moreover, they have an increased thymic repertoire, as well as an increased adaptive and innate immune response, characteristics which, at the outset, could protect them from COVID-19 compared to adults with the same characteristics [16]. They also show decreased ACE2 receptors on Type 2 pneumocytes, as well as high eosinophil concentrations, all of which are associated with a better prognosis of SARS-CoV-2 disease [21,23,30,35]. Two studies show that constant exposure to allergens and the consequent allergic sensitisation of asthmatic children also decrease these receptors [16,28]. In one of the studies, it is noted that the production of mucus and glycoproteins such as Muc5ac could prevent SARS-CoV-2 from reaching the distal airway and causing pathology [28,42].

Furthermore, Type 2 inflammation or Th2 phenotype (present in 50% of asthmatics) appears to protect against sepsis in experimental models and is therefore regarded as another possible cause of this protection against COVID-19 [21,22,28,31,43]. In one study, a decrease in the severity of COVID-19 disease was also observed in those with this phenotype [21]. Although excessive Type 2 inflammation facilitates viral-induced asthma exacerbations, it appears to be a protective element against this virus [21]. However, one of the studies rejects this hypothesis, as it reports increased mortality in this group [24].

### 3.6. Pharmacological Treatment

Inhaled corticosteroids are used in 75% of patients in China, a country with a low prevalence of asthmatics with COVID-19 [23].

Combined pharmacological treatment with nebulised budesonide, systemic steroids and nebulised salbutamol improves spirometric indices in the paediatric asthma population [43]. According to the Severe Asthma Research Program-3, inhaled corticosteroid treatment has been shown to decrease the number of ACE2 receptors and TMPRSS2 gene expression in sputum, both of which are associated with a reduced likelihood of SARS-CoV-2 entry through the airway [22,42]. Moreover, the combination of inhaled corticosteroids with bronchodilators in vitro decreases coronavirus replication and cytokine production [23]. Preliminary studies with budesonide also show that this combination reduces SARS-CoV-2 DNA replication and inhibits its cytopathic activity, which has significant clinical implications [22]. Inhaled ciclesonide, on the other hand, suppresses SARS-CoV-2 replication in vitro [34]. However, no relationship has been found between the dose of corticosteroids and the severity of COVID-19 disease [21], while systemic corticosteroids have been associated with worse prognoses, increased hospitalisations, and a higher viral load [22,30]. However, these treatments are recommended for severe asthma exacerbations unresponsive to bronchodilators [32,34]. Schultze et al. [37] reported increased mortality in patients treated with inhaled corticosteroids, associated with confounding variables. Moreover, the increase in therapeutic adherence (to which families have contributed significantly) is particularly beneficial in these cases due to its role in protecting against COVID-19 [20,22,25,34].

## 4. Discussion

The present systematic review has found evidence to support several conclusions. Most of the studies analysed present statistical data confirming a decrease in the incidence of COVID-19 in children and adolescents with asthma. The primary factors that could underlie this decrease are related to safety measures, asthma phenotypes and to inhaled corticosteroids. First, safety measures have a very important role on the trans-mission of this virus. Lockdown and the decrease of use of cars and other means of transport have resulted in a betterqualityair and a reduction of the number of asthma exacerbations in children and a decrease in hospital admissions [20]. Furthermore, the fact that children had to stay at their homes has also played an important role in limiting the viral transmission among children [20,25,27,29,31]. It is also perceived that the home exposure of these children to tobacco can increase exacerbations, as a negative consequence of lockdown, but this remains unclear [25,28,32,37]. However, confinement may cause psychological stress when following these extremely strict safety measures, that are not justified in the case of the asthmatic children [29,33,34]. Moreover, it is crucial to consider social factors such as the low socio-economic status of families, or malnutrition, both of which are linked to an increase in COVID-19 infections [25,27,34]. It is therefore essential that health care policies are focused on these social groups to address these gaps and to educate parents properly in order to achieve a better global health. Regarding asthmatic children characteristics and asthma phenotypes, several conclusions must be mentioned. Children have a lower concentration of ACE2 receptors and a high eosinophil concentration, which that could further contribute to protection against a COVID-19 infection [21,23,30,35]. Sensitisation to allergens among asthmatic children appears to de-crease the number of ACE2 receptors present in the airway [16,28,42]. Glycoproteins in the mucus such as Muc5ac play a role in making more difficult to COVID-19 to arrive to the distal airway and to cause an infection [28]. The presence of Type 2 inflammatory response could also be a protective factor due to an increased presence of cytokines [21]. These factors may be a cause of the milder course of the COVID-19 infection in children and also of approximately a 79% of the asymptomatic infections among them [7]. It is also important to note that the increase in therapeutic adherence has played a particularly relevant role in the lower incidence of COVID-19 in children and adolescents with asthma. Further, inhaled corticosteroids have increasingly shown to be beneficial when used to prevent a COVID-19 infection. Inhaled corticosteroid treatment has been shown to decrease the number of ACE2 receptors and TMPRSS2 gene expression in sputum, reducing SARSCov-2 entry through the airway [22,42]. Indeed, the combination of bronchodilators and inhaled corticosteroids has shown to decrease coronavirus replication and cytokine production [23]. However, no relationship has been found between the dose of corticosteroids and the severity of COVID-19 disease [21]. Systemic corticosteroids, on the other hand, have been associated with a worse prognosis, increased hospitalisations, and a higher viral load [22,30], leaving the use of this treatment only for severe asthma exacerbations that are unresponsive to bronchodilators [32,34]. Nevertheless, this evidence should be further analysed in future works, since many of the studies included in this review were conducted in vitro. All in all, as we have shown in this paper, there are many factors that contribute to protect asthmatic children against COVID-19 infection but, we have to keep in mind that, even though is not possible yet due to the lack of clinical trials on children, we will only reach their protection and immunity through the development of a vaccine for them [7]. This is especially important regarding the school return, due to the easy transmission of the virus in this environment. Also, stay at home orders have had a substantial impact on children and young people, including decreased vaccinationrates, delayed management of health conditions, prolonged exposure to in-door home air pollutants, and impacts on mental health, it is possible that in some children and young people a return to school might improve overall asthma control [44]. Finally, it should be noted that only a limited number of studies have been performedin the paediatric population, an issue that should be addressed since this is a source of bias in the study data. Moreover, the gender perspective has been understudied in this age group, probably due to the scarce information of children concerning this topic.

## 5. Conclusions

Scientific evidence suggests that the infant and juvenile population is protected against COVID-19 due to several factors. Safety measures are shown to be very effective in protecting against SARS-CoV-2 [20,25,27,29,31]. Based on the scientific evidence presented here, it is recommended to follow current treatment guidelines. We also recommend continuing the treatment with inhaled corticosteroids due to its benefits shown in the studies cited [22,23,42]. Systemic corticosteroids should be left just for severe asthma exacerbations with no response to bronchodilators, as they lead to an increase of the hospitalisation rate and viral load [22,30]. Besides, children seem to have unique characteristics, such as a lower concentration of ACE2 receptors or an increase in eosinophils, that protect them against the COVID-19 infection [21,23,30,35]. Particularly in asthmatic children, sensitisation to allergens, and also glycoproteins in mucus such as MUC5ac and type 2 inflammatory response are factors that protect these children against SARS-CoV-2 [16,21,28,42]. It is also very important to educate patients and families during visits to health centers, to provide the knowledge and skills necessary to improve care in daily life and the skills needed to improve their autonomy and therapeutic compliance. In addition, it is essential to coordinate the professionals from Primary Care and Specialised Care, with patients and their families [10]. However, despite these findings, ongoing research in this field is needed to address unanswered questions, particularly concerning children, a population with a high prevalence of asthma. Unfortunately, the dramatic decline inasthma morbidity is believed to have an impact on ongoing asthma clinicalintervention trials relying on exacerbations as a primary outcome, as it has been noticed in the USA [45]. Furthermore, some countries’ low asthma prevalence, such as China, may also difficult the possibility of effective clinical trials [46].We encourage introduction of the gender perspective, not so frequently seen, and studies in all age groups. If we continue along this path, we will be able to change all these children’s lives and, moreover, we will be able to change the way we perceive chronic diseases such as asthma. Children are one of the groups most affected by the lockdown period, so the better we understand this situation, the better the life of these children will be.

## Figures and Tables

**Table 1:** Summary ofsystematic review results.

Name	Country	Date of Publication	Type of Study	Variables/Objectives	Results
Ciprandi G et al., 2020 [16]	Italy	September 2020	Letter to the Editor	n.s. ^a^	Decreased prevalence of asthma in patients with COVID-19.
Krivec U et al., 2020 [20].	Slovenia	August 2020	Observational data analysis	Admissions for asthma andrespiratory infections, NO_2_ concentration and concentration of particulate matter in the air.	A decrease in the number of admissions for asthma and respiratory infections than other years in children is attributable to improved air quality due to confinement and compliance with safety measures. None of the children admitted were COVID19 positive.
Muñoz X et al., 2020 [21].	Spain	n.s. ^a^	Letter to the Editor	n.s. ^a^	Asthma is not associated with the risk of contracting COVID-19. No relationship is found between the dose of inhaled corticosteroids and COVID-19 severity. A decrease in the severity of COVID-19 disease is observed in patients with a Th2 phenotype.
Morais Almeida M et al., 2020 [22]	n.s. ^a^	June 2020	Systematic review	In patients with SARS-CoV-2: Asthma, comorbidities, hospitalisation, epidemiology.	Asthmatic children are not at risk for SARS-CoV-2. Inhaled corticosteroids are beneficial in protecting against the current coronavirus, as opposed to systemic corticosteroids. Th2 inflammation may be related to a better prognosis.
Halpin DMG and Cols, 2020 [23]	n.s. ^a^	April 2020	Observational data analysis	Prevalence of asthmatic patients with COVID, inhaled corticosteroid use.	The underdiagnosis hypothesis and the modified immune response hypothesis are rejected in favour of inhaled corticosteroids.
Castro Rodriguez JA et al., 2020 [24]	n.s. ^a^	June 2020	Systematic review	SARS-CoV-2, wheezing, asthma, children (0–18 years).	We identify an underrepresentation of children in the studies reviewed. Asthmatics could be protected against COVID for various reasons: inhaled corticosteroids, changes in the immune response, or underdiagnosis of this pathology.
Oreskovic NM et al., 2020 [25]	United States	May 2020	n.s. ^a^	ED visits by paediatric asthmatic patients, air quality, therapeutic adherence.	Decrease in emergency room visits of paediatric asthmatic patients.
Chavasse RJ et al., 2020 [26]	United Kingdom	July 2020	Letter to the Editor	n.s. ^a^	Decreased asthmatic attendance to emergency departments due to therapeutic adherence, improved air quality, and confinement.
To T et al., 2020 [27]	n.s. ^a^	June 2020	n.s. ^a^	Proposed actions to address COVID-19 for people with respiratory pathologies.	Decrease in Prevalence ofCOVID19in asthmatics. Proposal for improving the health care of this group.
Farne H et al., 2020 [28]	United Kingdom	n.s. ^a^	Letter to the Editor	n.s. ^a^	Asthmatic children have a specific phenotype that protects from SARS-CoV-2.
Papadopoulos NG et al., 2020 [29]	n.s. ^a^	September 2020	Online survey	To describe the impact of COVID-19 on asthmatic patients and hospital services.	Asthmatic children are notdisproportionately affected. No support for stringent confinement measures in this population.
Creese H y cols, 2020 [30]	United Kingdom	November 2020	n.s. ^a^	n.s. ^a^	In favour of inhaled corticosteroids. Rhinoviruses associated with the majority of asthmatic exacerbations.
Hanon S et al., 2020 [31]	Belgium	n.s. ^a^	Observational study	To evaluate the incidence of COVID-19 in patients with severe asthma.	Asthmatic patients are not at risk of SARS-CoV2.
Hepkaya E et al., 2020 [32]	Turkey	September 2020	Population survey	To assess the current status of asthmatics during the pandemic.	Evidence in favour of decreased prevalence of COVID19 in the asthmatic population and the beneficial use of inhaled corticosteroids.
Kabesch M, 2020 [33]	Germany	July 2020	Letter to the Editor	n.s. ^a^	There is no justification for extreme safety measures in asthmatic children.
Abrams EM et al., 2020 [34]	n.s. ^a^	September 2020	State-of-the-art	Paediatric asthma review and COVID-19.	A lower concentration of ACE2 receptors protects asthmatics. Impact of socialdeterminants. Support for treatment with inhaled corticosteroids. Extreme safety measures are not justified.
Camiolo M et al., 2020 [35]	United States	May 2020	Cohort Study	Use of various clinical parameters relevant to the study of ACE2 receptors.	Increased ACE2 receptors are associated with a worse prognosis.
Ruano FJ et al., 2020 [36]	Spain	October 2020	n.s. ^a^	n.s. ^a^	Asthmatic children have milder forms of COVID-19 disease.
Schultze A et al., 2020 [37]	United Kingdom	September 2020	Cohort Study	Patients with asthma or COPD, treatment with corticoids or longacting Beta agonists or LAMA/LABA.	Increased mortality was found in patients taking inhaled corticosteroids, attributed to confounding variables.

^a^ n.s.: not specified.The data shown in the table are grouped into four dimensions, described below.

**Table 2:** Prevalence of paediatric asthma in COVID-19 studies.

Location/Name of Study	Country	Results
South Lombardy and Liguria Hospitals [16].	Italy	Of 52 paediatric patients, only 1 had asthma (2%).
Ljubljana Children’s Hospital [20]	Slovenia	71% to 78% decrease in asthma admissions compared to previous years.
Servei de Pneumologia Hospital Valld’Hebron [21]	Spain	Of 2226 hospitalised patients, only 3.2% were asthmatic.
Children’s Hospital, Wuhan [24]	China	Of 171 paediatric patients with COVID, none were asthmatic.
Hubei Province [40]	China	Of 25 paediatric patients with COVID, none were asthmatic.
Confidence Study [38]	Italy	Of 100 paediatric patients with COVID, none were asthmatic.
COVID-19 in Children [41]	USA	Asthma was the most frequent comorbidity.
Massachusetts General Hospital [8,25]	USA	The number of emergency department visits by asthmatic patients decreased by 38.8% and 84.8% during March and April 2020, respectively.
St George’s University Hospitals NHS Foundation Trust [26]	United Kingdom	90% decrease in asthma admissions.

## Data Availability

Not applicable.
